# Crystal structure of *r*-1,*c*-2-dibenzoyl-*t*-3,*t*-4-bis­(2-nitro­phen­yl)cyclo­butane

**DOI:** 10.1107/S2056989017015936

**Published:** 2017-11-17

**Authors:** Manuel Velasco Ximello, Sylvain Bernès, Aarón Pérez-Benítez, Ulises Hernández Pareja, Angel Mendoza, Jorge R. Juárez Posadas, Jaime Vázquez Bravo

**Affiliations:** a Centro de Química, Instituto de Ciencias, Benemérita Universidad Autónoma de Puebla, Edif. IC8 Complejo de Ciencias, C.U., 72570 Puebla, Pue., Mexico; b Instituto de Física, Benemérita Universidad Autónoma de Puebla, Av. San Claudio y 18 Sur, 72570 Puebla, Pue., Mexico; c Facultad de Ciencias Químicas, Benemérita Universidad Autónoma de Puebla, Av. San Claudio y 18 Sur, 72570 Puebla, Pue., Mexico; d Ingeniería en Biotecnología, Universidad Politécnica Metropolitana de Puebla, Popocatépetl s/n, Tres Cerritos, 72480 Puebla, Pue., Mexico

**Keywords:** crystal structure, cyclo­butane, truxillic isomer, truxinic isomer, chalcone

## Abstract

The condensation reaction of aceto­phenone (1-phenyl­ethan-1-one) with 2-nitro­benzaldehyde in the molten state yielded the expected chalcone, (*E*)-3-(2-nitro­phen­yl)-1-phenyl­prop-2-en-1-one, and, unexpectedly, the title compound, C_30_H_22_N_2_O_6_, which results from the *syn* head-to-head [2 + 2] cyclo­addition of the chalcone. The mol­ecular structure of the dimer shows that the four benzene rings of the substituents are oriented in such a way that potential steric hindrance is minimized, whilst allowing some degree of inter­molecular π–π inter­actions for crystal stabilization. In the mol­ecule, one nitro group is disordered over two positions, with occupancies for each part of 0.876 (7) and 0.127 (7).

## Abstract 2

The title mol­ecule is a tetra­substituted truxinic-type cyclo­butane derivative with a central ring that is almost planar despite of being placed in a general position. The mol­ecular structure of the dimer shows that the four benzene rings of the substituents are oriented in such a way that potential steric hindrance is minimized, whilst allowing some degree of inter­molecular π–π inter­actions for crystal stabilization.

## Chemical context   

The [2 + 2]-photo­cyclo­addition reaction is the most frequently used photochemical reaction to access four-membered carbon rings. An emblematic application of this large class of reactions is the synthesis of cage compounds such as cubane (Eaton & Cole, 1964 ▸). On the other hand, [2 + 2] cyclo­addition may be also a key tool for the synthesis of some natural compounds including a functionalized cyclo­butane ring, for example sceptrin, isolated from a marine sponge (Ma *et al.*, 2014 ▸), ediandrin, isolated from the roots of an Australian rainforest plant (Davis *et al.*, 2009 ▸), or incarvillateine, isolated from the aerial parts of a wild plant found in China (Nakamura *et al.*, 1999 ▸; Ichikawa *et al.*, 2004 ▸).
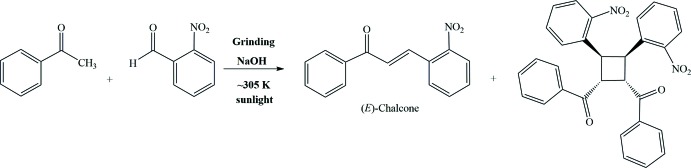


**Figure 1:**
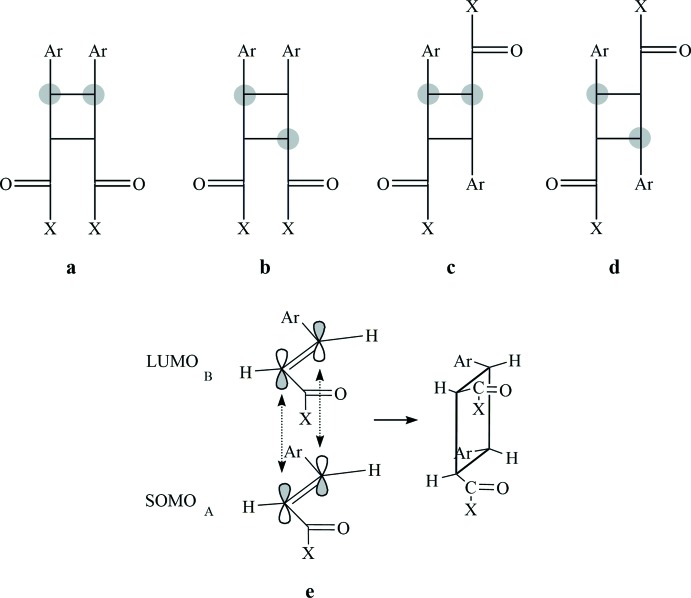
The four possible stereoisomers resulting from the [2 + 2]-cyclo­addition of (*E*)-chalcones (Cibin *et al.*, 2003 ▸): (*a*) *syn* head-to-head; (*b*) *anti* head-to-head; (*c*) *syn* head-to-tail and (*d*) *anti* head-to-tail; (*e*) *supra*–*supra* bonding inter­action (π^2^s + π^2^s) of SOMO–LUMO to produce the *syn* head-to-head stereoisomer shown in (*a*).

The syntheses of these compounds generally involves photochemical dimerization of olefins, α,β-unsaturated carbonyl, or carboxyl compounds. Traditionally, these compounds have been synthesized through inter­molecular [2 + 2]-photo­cyclo­addition reaction of 1,3-di­aryl­prop-2-en-1-ones (also known as chalcones) in solution (Kumar *et al.*, 2017 ▸), or in the solid state and molten state, under UV irradiation. The cyclo­addition of (*E*)-chalcones may give four possible stereoisomers, namely *syn*/*anti*, head-to-head and head-to-tail (Fig. 1 ▸), depending on the physical state of the substrate (solid, solution or molten state) and on other reaction conditions, such as the type of glassware used for the workup.

Herein we report the synthesis and structure of a new chalcone dimer, obtained fortuitously while preparing the monomeric chalcone (see *Scheme*). The title compound corresponds to the *syn* head-to-head stereoisomer (Fig. 1 ▸ *a*), which could arise from a *supra*–*supra* bonding inter­action between the singly occupied mol­ecular orbital (SOMO) of one chalcone and the lowest unoccupied mol­ecular orbital (LUMO) of the other one (Fig. 1 ▸ *e*; Smith, 2016 ▸). Since we detected only one stereoisomer corresponding to the cyclo­addition product in the mixture of the reaction carried out under sunlight, we assume that dimerization is actually performed *via* this mechanism. Indeed, the proposed mechanism is consistent with the structure reported herein.

## Structural commentary   

**Figure 2:**
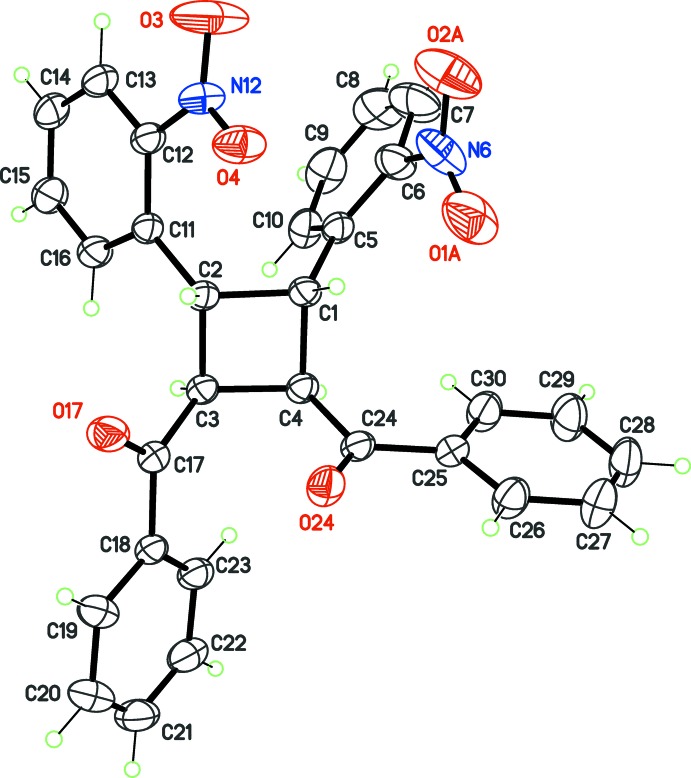
Mol­ecular structure of the title cyclo­butane, with displacement ellipsoids at the 30% probability level for non-H atoms. Disordered atoms O1*B* and O2*B* have been omitted.

The topochemical solid-state dimerization of the chalcone (*E*)-3-(2-nitro­phen­yl)-1-phenyl­prop-2-en-1-one resulted in the title tetra­substituted cyclo­butane derivative (Fig. 2 ▸). The *rctt* (*cis*, *trans*, *trans*) relative stereochemistry of the substituents is identical to that of β-truxinic acid, obtained by photodimerization of cinnamic acid (Hein, 2006 ▸), indicating that dimerization occurred *via* a *syn* head-to-head [2 + 2] cyclo­addition of the chalcone.

The mol­ecule potentially belongs to the *C_s_* point group, but crystallizes in a general position in space group *P* 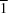. The cyclo­butane ring is thus non-planar, unlike many head-to-tail photodimerizations adducts, which crystallize with the ring placed about an inversion centre (see *Database survey* section). However, the departure from planarity is very small, the dihedral angle between the C1/C2/C3 and C1/C4/C3 mean planes being 3.6 (2)°. Some other *rctt* tetra­substituted cyclo­butane derivatives have a more marked butterfly conformation, which apparently results from steric restrictions imposed by bulky substituents (*e.g*. Strabler *et al.*, 2013 ▸). In the case of the title compound, the *cis* benzoyl and nitro­benzene groups are oriented in such a way that intra­molecular π–π or C—H⋯π contacts are avoided. The shortest centroid-to-centroid separation is larger than 4.2 Å, for the nitro­benzene rings, which form a dihedral angle of 45.73 (8)°. In contrast, an inter­molecular π–π contact is formed by parallel C11–C16 mnito­benzene rings related by an inversion centre. In that case, the separation between the rings is 3.883 (1) Å. These features seem to indicate that the mol­ecular conformation is optimized in order to avoid steric hindrance, whilst at the same time allowing an efficient packing for the crystal stabilization.

The geometry of the cyclo­butane ring matches the statistics computed by *MOGUL* (Bruno *et al.*, 2004 ▸). The C—C bond lengths range from 1.542 (2) to 1.580 (3) Å and the C—C—C angles range from 88.90 (13) to 90.76 (13)° (*MOGUL* medians: *m* = 1.558–1.565 Å and *m* = 88.7–89.5°, respectively). On the other hand, the average of absolute values for torsion angle defined by the four C atoms of the cyclo­butane ring is 〈|δ|〉 = 2.52 (2)°. All these features support the conclusion reached by another research group who determined the structure of a closely related compound, namely a cyclo­butane substituted by two benzoyl and two meth­oxy­phenyl groups (Steyl *et al.*, 2005 ▸): the total distortion of the cyclo­butane ring increases while additional functionalization of the benzene rings is achieved, due mainly to steric effects. In that sense, the title mol­ecule belongs to the class of cyclo­butane derivatives exhibiting almost no puckering distortion.

## Database survey   

A survey of the current organic sub-set of the CSD database (CSD 5.38 updated May 2017; Groom *et al.*, 2016 ▸) was performed for cyclo­butane derivatives formulated C_4_H_4_ *R*_2_ *R*′_2_ where *R* and *R*′ are two different substituents. The data set was limited to cyclo­butanes for which each C atom is substituted by exactly one H atom and one non-H substituent, and all hits for which the cyclo­butane is fused with one or various cyclic systems were omitted. The resulting hits for which 3D coord­inates are available were checked by hand in order to eliminate cyclo­butanes substituted by three or four different substituents and those for which the four substituents are identical. Finally, structures determined several times were filtered to avoid statistical bias, and some severely disordered cases unsuitable for geometric computations were also deleted. The final working set contained 225 cyclo­butanes C_4_H_4_ *R*_2_ *R*′_2_ comparable with the title compound (see deposited *Excel* file).

**Figure 3:**
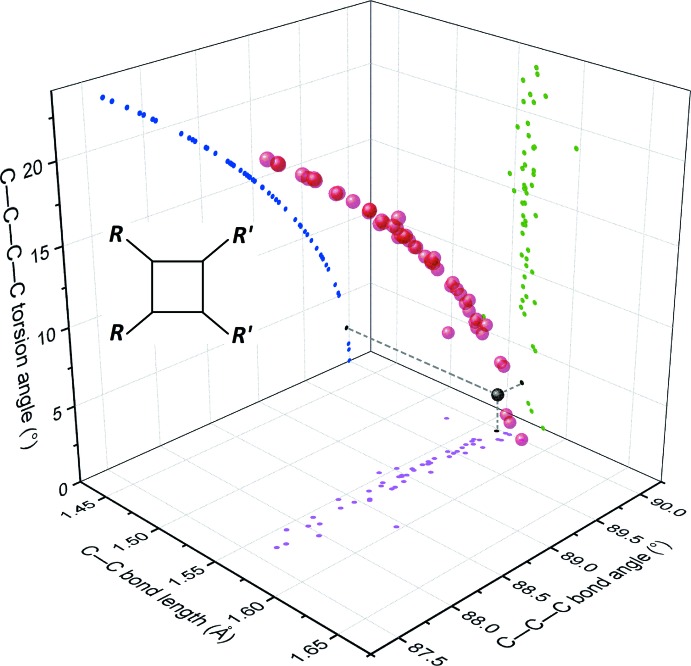
A plot of the geometric parameters for truxinic-type cyclo­butanes with formula C_4_H_4_ *R*_2_ *R*′_2_ retrieved from the CSD. Each red ball corresponds to one cyclo­butane ring in the three-dimensional space defined by the average of four bond lengths, the average of four bond angles, and the average of the absolute values of the four torsion angles in the cycle. This distribution is also projected on three two-dimensional spaces, for each couple of geometric parameters (magenta, green, and blue dots). The parameters for the title compound are represented with black dots.

**Figure 4:**
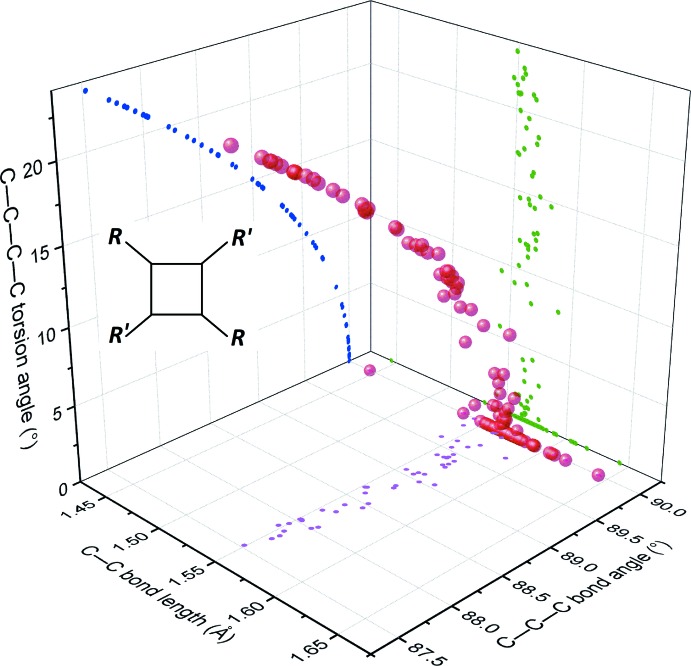
A plot similar to that of Fig. 3 ▸, for truxillic-type cyclo­butanes with formula C_4_H_4_ *R*_2_ *R*′_2_ retrieved from the CSD. The same scale for the three axis of the space is used in both figures, for comparison purposes. Note the cluster of points with the averages of bond angles constrained to 90°, corresponding to cyclo­butanes planar by symmetry.

Within this set, 77% of the cyclo­butanes result formally from a head-to-tail dimerization (known as truxillic type), many of them (108) with the cyclo­butane lying on a special position. The remaining 23% result formally from a head-to-head dimerization (known as truxinic type), and only one of them displays crystallographic symmetry (cyclo­butane of sceptrin, placed on a twofold axis in space group *C*2; Ma *et al.*, 2014 ▸). Although the truxillic cyclo­butanes thus have a marked tendency to be more ‘symmetric’ than the truxinic co-set, both groups are very similar regarding their conformational flexibility. The range of distortion accessible for the cyclo­butane ring may be estimated by plotting the geometric parameters describing the conformation of the ring: bond lengths, angles, and torsion angles (Figs. 3 ▸ and 4 ▸). The distributions observed for 52 truxinic cyclo­butanes (Fig. 3 ▸) and 174 truxillic cyclo­butanes (Fig. 4 ▸) are almost identical, with the exception of the accumulation of data at bond angles of 90° in the latter, due to the occurrence of rings planar by symmetry. The same applies to the distortion of the rings in both groups, for example, for the functions τ = τ(θ), where θ is a bond angle and τ a torsion angle (blue curves in Figs. 3 ▸ and 4 ▸). Indeed, these distributions are perfectly fitted using the same power function in both groups: τ = 15(θ − 90)^0.5^, where τ and θ are expressed in degrees.

The title compound belongs to the truxinic group, and exhibits a very small distortion for the cyclo­butane ring, compared to other truxinic derivatives (see black dots in Fig. 3 ▸). Only three other related truxinic derivatives for which the X-ray structures have been published present a more planar cyclo­butane ring. It thus appears that the substituents in the title mol­ecule, benzoyl and 2-nitro­phenyl, have very little steric influence on the central ring.

## Synthesis and crystallization   

A mixture of aceto­phenone (0.52 g, 4.38 mmol), 2**-**nitro­benzaldehyde (0.66 g, 4.38 mmol) and solid NaOH pellets (0.17 g, 4.38 mmol) were ground in an agate mortar with a pestle, at room temperature, for 23 min. The reaction proceeds exothermically (as noted by a rise in temperature of about 5–12 K). The progress of the reaction was monitored by TLC. After completion, the mixture was diluted with CH_2_Cl_2_ and washed with brine. The organic layer was separated, dried over MgSO_4_ and evaporated under reduced pressure. The crude product was purified by column chromatography using silica-gel and hexa­nes–ethyl acetate 4:1 as eluent, to give the expected chalcone and the title compound (0.66 g, 30%), as brown and colourless solids, respectively. Cyclo­butane deriv­ative: m.p. 517 K. FT–IR ν_max/_cm^−1^ 1659 (C=O), 1557, 1348 (NO_2_). ^1^H NMR (500 MHz, CDCl_3_) δ/ppm: 7.81–7.33 (18H, *m*, ArH), 5.22 (2H, *m*, CH) and 4.87 (2H, *m*, CH). ^13^C NMR (125 MHz, CDCl_3_) δ/ppm: 44.3, 46.8, 125.0, 128.3, 129.4, 129.6, 130.4, 133.1, 134.7, 136.7, 137.5, 148.6, 196.9. HRMS (EI) calculated for C_30_H_22_N_2_O_6_ (*M* ^+^) 506.1478; found 506.1477.

## Refinement   

Crystal data, data collection and structure refinement details are summarized in Table 1 ▸. One of the nitro groups is disordered by rotation about its C—NO_2_ bond, and was refined with two parts for the O atoms: O1*A*/O2*A* with occupancy 0.876 (7) and O1*B*/O2*B* with occupancy 0.124 (7). These four sites were restrained to have similar displacement parameters, with standard deviation of 0.04 Å^2^. The same restriction was applied to the O atoms of the other nitro group, O3/O4, given that this nitro group is probably also affected by disorder, although we were unable to refine a suitable model on the basis of the room temperature data for this group. The C-bound H atoms were treated as riding atoms in geometrically idealized positions: C—H 0.93–0.98 Å with *U*_iso_(H) = 1.2*U*_eq_(C).

## Supplementary Material

Crystal structure: contains datablock(s) I, global. DOI: 10.1107/S2056989017015936/ex2002sup1.cif

Structure factors: contains datablock(s) I. DOI: 10.1107/S2056989017015936/ex2002Isup2.hkl

excel file for statistic analysis. DOI: 10.1107/S2056989017015936/ex2002sup3.txt

Supporting information file. DOI: 10.1107/S2056989017015936/ex2002Isup4.cml

CCDC reference: 1583527

Additional supporting information:  crystallographic information; 3D view; checkCIF report

## Figures and Tables

**Table 1:** Experimental details

Crystal data	
Chemical formula	C_30_H_22_N_2_O_6_
*M*_r_	506.49
Crystal system, space group	Triclinic, *P* 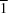
Temperature (K)	295
*a*, *b*, *c* (Å)	7.2599 (5), 10.5614 (5), 16.7351 (8)
α, β, γ (°)	78.863 (4), 87.472 (5), 85.238 (5)
*V* (Å^3^)	1254.13 (13)
*Z*	2
Radiation type	Mo *K*α
μ (mm^−1^)	0.10
Crystal size (mm)	0.37 × 0.20 × 0.15

Data collection	
Diffractometer	Agilent Xcalibur Atlas Gemini
Absorption correction	Analytical (*CrysAlis PRO*; Agilent, 2013 ▸)
*T*_min_, *T*_max_	0.962, 0.983
No. of measured, independent and observed [*I* > 2σ(*I*)] reflections	25649, 5116, 3292
*R*_int_	0.051
(sin θ/λ)_max_ (Å^−1^)	0.625

Refinement	
*R*[*F* ^2^ > 2σ(*F* ^2^)], *wR*(*F* ^2^), *S*	0.053, 0.164, 1.04
No. of reflections	5116
No. of parameters	363
No. of restraints	24
H-atom treatment	H-atom parameters constrained
Δρ_max_, Δρ_min_ (e Å^−3^)	0.27, −0.27

Computer programs: *CrysAlis PRO* (Agilent, 2013 ▸), *SHELXT2014* (Sheldrick, 2015*a* ▸), *SHELXL2016* (Sheldrick, 2015*b* ▸), *XP* in *SHELXTL* (Sheldrick, 2008 ▸) and *Mercury* (Macrae *et al.*, 2008 ▸).

## supplementary crystallographic information

### Crystal data

**Table:**

C_30_H_22_N_2_O_6_	*F*(000) = 528
*M**_r_* = 506.49	*D*_x_ = 1.341 Mg m^−^^3^
Triclinic, *P*1	Melting point: 517 K
*a* = 7.2599 (5) Å	Mo *K*α radiation, λ = 0.71073 Å
*b* = 10.5614 (5) Å	Cell parameters from 5272 reflections
*c* = 16.7351 (8) Å	θ = 3.6–27.9°
α = 78.863 (4)°	µ = 0.10 mm^−^^1^
β = 87.472 (5)°	*T* = 295 K
γ = 85.238 (5)°	Prism, colourless
*V* = 1254.13 (13) Å^3^	0.37 × 0.20 × 0.15 mm
*Z* = 2	

### Data collection

**Table:**

Agilent Xcalibur Atlas Gemini diffractometer	5116 independent reflections
Radiation source: Enhance (Mo) X-ray Source	3292 reflections with *I* > 2σ(*I*)
Graphite monochromator	*R*_int_ = 0.051
Detector resolution: 10.5564 pixels mm^-1^	θ_max_ = 26.4°, θ_min_ = 3.1°
ω scans	*h* = −9→8
Absorption correction: analytical (CrysAlis PRO; Agilent, 2013)	*k* = −13→13
*T*_min_ = 0.962, *T*_max_ = 0.983	*l* = −20→20
25649 measured reflections	

### Refinement

**Table:**

Refinement on *F*^2^	Secondary atom site location: difference Fourier map
Least-squares matrix: full	Hydrogen site location: inferred from neighbouring sites
*R*[*F*^2^ > 2σ(*F*^2^)] = 0.053	H-atom parameters constrained
*wR*(*F*^2^) = 0.164	*w* = 1/[σ^2^(*F*_o_^2^) + (0.0762*P*)^2^ + 0.1873*P*] where *P* = (*F*_o_^2^ + 2*F*_c_^2^)/3
*S* = 1.04	(Δ/σ)_max_ < 0.001
5116 reflections	Δρ_max_ = 0.27 e Å^−^^3^
363 parameters	Δρ_min_ = −0.27 e Å^−^^3^
24 restraints	Extinction correction: SHELXL2016 (Sheldrick, 2015b), Fc^*^=kFc[1+0.001xFc^2^λ^3^/sin(2θ)]^-1/4^
0 constraints	Extinction coefficient: 0.011 (3)
Primary atom site location: structure-invariant direct methods	

### Fractional atomic coordinates and isotropic or equivalent isotropic displacement parameters (Å^2^)

**Table:**

	*x*	*y*	*z*	*U*_iso_*/*U*_eq_	Occ. (<1)
C1	0.6552 (3)	0.64030 (16)	0.23390 (11)	0.0415 (4)	
H1A	0.548307	0.694892	0.209849	0.050*	
C2	0.6594 (3)	0.63982 (16)	0.32828 (11)	0.0406 (4)	
H2A	0.558141	0.699162	0.342918	0.049*	
C3	0.8388 (3)	0.71065 (17)	0.31510 (11)	0.0415 (4)	
H3A	0.942691	0.650434	0.336831	0.050*	
C4	0.8326 (3)	0.71816 (17)	0.22142 (11)	0.0414 (4)	
H4A	0.938656	0.668933	0.200920	0.050*	
C5	0.6680 (3)	0.51217 (17)	0.20721 (11)	0.0466 (5)	
C6	0.5325 (3)	0.4711 (2)	0.16339 (15)	0.0648 (6)	
C7	0.5406 (5)	0.3472 (3)	0.1475 (2)	0.0961 (10)	
H7A	0.447643	0.322545	0.118570	0.115*	
C8	0.6849 (5)	0.2614 (3)	0.1744 (2)	0.0980 (10)	
H8A	0.690019	0.177555	0.164369	0.118*	
C9	0.8229 (5)	0.2982 (2)	0.21645 (17)	0.0815 (8)	
H9A	0.922486	0.239823	0.234101	0.098*	
C10	0.8140 (3)	0.4216 (2)	0.23247 (13)	0.0591 (6)	
H10A	0.908604	0.445158	0.261050	0.071*	
C11	0.6561 (3)	0.51184 (17)	0.38742 (11)	0.0424 (4)	
C12	0.5073 (3)	0.43324 (18)	0.39869 (12)	0.0466 (5)	
C13	0.5125 (3)	0.3138 (2)	0.45159 (13)	0.0564 (6)	
H13A	0.410934	0.264323	0.456958	0.068*	
C14	0.6658 (4)	0.2693 (2)	0.49537 (13)	0.0637 (6)	
H14A	0.670172	0.189494	0.530677	0.076*	
C15	0.8138 (3)	0.3437 (2)	0.48669 (14)	0.0646 (6)	
H15A	0.919549	0.314127	0.516170	0.078*	
C16	0.8071 (3)	0.4627 (2)	0.43438 (13)	0.0565 (6)	
H16A	0.908810	0.511744	0.430641	0.068*	
C17	0.8343 (3)	0.83090 (18)	0.35266 (11)	0.0449 (5)	
C18	0.9976 (3)	0.91010 (17)	0.34225 (11)	0.0448 (5)	
C19	0.9958 (3)	1.0116 (2)	0.38363 (15)	0.0633 (6)	
H19A	0.892064	1.031224	0.414871	0.076*	
C20	1.1465 (4)	1.0838 (3)	0.37887 (18)	0.0789 (8)	
H20A	1.144193	1.151593	0.407100	0.095*	
C21	1.3000 (4)	1.0563 (3)	0.33276 (17)	0.0766 (8)	
H21A	1.401455	1.105457	0.329712	0.092*	
C22	1.3038 (3)	0.9565 (2)	0.29124 (15)	0.0642 (6)	
H22A	1.407992	0.937882	0.259970	0.077*	
C23	1.1527 (3)	0.8830 (2)	0.29563 (13)	0.0527 (5)	
H23A	1.155681	0.815337	0.267201	0.063*	
C24	0.7924 (3)	0.84768 (18)	0.16696 (12)	0.0438 (5)	
C25	0.8062 (3)	0.85576 (19)	0.07748 (12)	0.0469 (5)	
C26	0.7499 (3)	0.9703 (2)	0.02607 (14)	0.0623 (6)	
H26A	0.703596	1.041174	0.048177	0.075*	
C27	0.7615 (4)	0.9808 (3)	−0.05686 (15)	0.0782 (8)	
H27A	0.723599	1.058504	−0.090560	0.094*	
C28	0.8287 (4)	0.8769 (3)	−0.09014 (15)	0.0817 (8)	
H28A	0.836448	0.884338	−0.146453	0.098*	
C29	0.8843 (4)	0.7627 (3)	−0.04106 (15)	0.0809 (8)	
H29A	0.929398	0.692340	−0.063924	0.097*	
C30	0.8737 (4)	0.7515 (2)	0.04271 (14)	0.0647 (6)	
H30A	0.911977	0.673403	0.075957	0.078*	
N6	0.3729 (4)	0.5581 (3)	0.13166 (19)	0.0906 (8)	
O1A	0.3951 (6)	0.6676 (4)	0.1010 (3)	0.1231 (16)	0.876 (7)
O2A	0.2222 (4)	0.5095 (3)	0.1392 (2)	0.1287 (16)	0.876 (7)
O1B	0.307 (5)	0.642 (4)	0.154 (3)	0.149 (13)	0.124 (7)
O2B	0.360 (5)	0.567 (3)	0.0504 (17)	0.209 (16)	0.124 (7)
N12	0.3353 (2)	0.47251 (18)	0.35551 (12)	0.0607 (5)	
O3	0.2211 (3)	0.3957 (2)	0.3593 (2)	0.1493 (13)	
O4	0.3067 (2)	0.58039 (15)	0.31765 (11)	0.0740 (5)	
O17	0.7042 (2)	0.85530 (15)	0.39699 (10)	0.0658 (5)	
O24	0.7394 (2)	0.94134 (13)	0.19653 (9)	0.0615 (4)	

### Atomic displacement parameters (Å^2^)

**Table:**

	*U*^11^	*U*^22^	*U*^33^	*U*^12^	*U*^13^	*U*^23^
C1	0.0485 (11)	0.0308 (9)	0.0445 (10)	−0.0046 (8)	−0.0076 (8)	−0.0030 (8)
C2	0.0457 (10)	0.0316 (9)	0.0440 (10)	−0.0048 (8)	−0.0007 (8)	−0.0055 (8)
C3	0.0470 (10)	0.0339 (10)	0.0421 (10)	−0.0059 (8)	−0.0030 (8)	−0.0023 (8)
C4	0.0483 (11)	0.0326 (10)	0.0421 (10)	−0.0035 (8)	0.0017 (8)	−0.0049 (8)
C5	0.0628 (13)	0.0345 (10)	0.0422 (11)	−0.0086 (9)	0.0004 (9)	−0.0051 (8)
C6	0.0732 (16)	0.0551 (14)	0.0720 (15)	−0.0143 (12)	−0.0027 (12)	−0.0229 (12)
C7	0.113 (2)	0.0699 (19)	0.122 (3)	−0.0273 (18)	−0.004 (2)	−0.0510 (18)
C8	0.141 (3)	0.0449 (15)	0.117 (3)	−0.0163 (18)	0.019 (2)	−0.0380 (16)
C9	0.120 (2)	0.0465 (14)	0.0735 (17)	0.0141 (14)	0.0103 (16)	−0.0105 (12)
C10	0.0822 (16)	0.0420 (12)	0.0506 (12)	0.0059 (11)	−0.0009 (11)	−0.0074 (9)
C11	0.0531 (11)	0.0351 (10)	0.0397 (10)	−0.0077 (8)	0.0028 (8)	−0.0076 (8)
C12	0.0520 (12)	0.0402 (11)	0.0480 (11)	−0.0092 (9)	0.0055 (9)	−0.0082 (8)
C13	0.0727 (15)	0.0432 (12)	0.0525 (12)	−0.0185 (11)	0.0069 (11)	−0.0035 (9)
C14	0.0940 (18)	0.0417 (12)	0.0511 (13)	−0.0135 (12)	−0.0039 (12)	0.0056 (10)
C15	0.0793 (16)	0.0530 (13)	0.0561 (13)	−0.0101 (12)	−0.0166 (11)	0.0084 (10)
C16	0.0667 (14)	0.0472 (12)	0.0531 (12)	−0.0158 (10)	−0.0126 (10)	0.0037 (9)
C17	0.0540 (12)	0.0406 (11)	0.0396 (10)	−0.0094 (9)	−0.0002 (9)	−0.0045 (8)
C18	0.0523 (11)	0.0374 (10)	0.0425 (10)	−0.0107 (9)	−0.0059 (9)	0.0019 (8)
C19	0.0747 (15)	0.0518 (13)	0.0689 (15)	−0.0207 (11)	0.0016 (12)	−0.0192 (11)
C20	0.094 (2)	0.0624 (16)	0.0895 (19)	−0.0351 (14)	−0.0022 (16)	−0.0237 (14)
C21	0.0775 (18)	0.0671 (17)	0.0843 (18)	−0.0349 (14)	−0.0142 (14)	0.0031 (14)
C22	0.0563 (13)	0.0607 (15)	0.0687 (15)	−0.0143 (11)	−0.0032 (11)	0.0093 (12)
C23	0.0547 (12)	0.0450 (12)	0.0552 (12)	−0.0096 (10)	−0.0055 (10)	0.0022 (9)
C24	0.0495 (11)	0.0356 (10)	0.0455 (11)	−0.0080 (8)	0.0035 (8)	−0.0047 (8)
C25	0.0509 (11)	0.0442 (11)	0.0444 (11)	−0.0103 (9)	0.0007 (9)	−0.0029 (9)
C26	0.0765 (15)	0.0528 (13)	0.0530 (13)	0.0000 (11)	−0.0017 (11)	−0.0003 (10)
C27	0.0956 (19)	0.0791 (18)	0.0502 (14)	0.0041 (15)	−0.0057 (13)	0.0078 (13)
C28	0.106 (2)	0.094 (2)	0.0416 (13)	−0.0032 (17)	−0.0048 (13)	−0.0059 (14)
C29	0.114 (2)	0.0783 (18)	0.0509 (14)	0.0014 (15)	0.0051 (14)	−0.0198 (13)
C30	0.0890 (17)	0.0526 (13)	0.0501 (13)	−0.0018 (12)	0.0024 (11)	−0.0066 (10)
N6	0.0822 (18)	0.0880 (19)	0.114 (2)	−0.0055 (17)	−0.0415 (15)	−0.0410 (19)
O1A	0.133 (3)	0.084 (2)	0.150 (4)	−0.005 (2)	−0.085 (3)	0.002 (2)
O2A	0.0728 (18)	0.148 (3)	0.183 (4)	−0.0225 (16)	−0.0267 (17)	−0.064 (2)
O1B	0.15 (2)	0.12 (2)	0.19 (3)	0.075 (19)	−0.081 (19)	−0.08 (2)
O2B	0.27 (3)	0.25 (3)	0.12 (2)	0.09 (2)	−0.13 (2)	−0.109 (19)
N12	0.0526 (11)	0.0481 (11)	0.0804 (13)	−0.0163 (9)	0.0023 (9)	−0.0054 (10)
O3	0.1017 (17)	0.0876 (15)	0.241 (3)	−0.0563 (14)	−0.0743 (18)	0.0496 (17)
O4	0.0612 (10)	0.0490 (10)	0.1066 (14)	−0.0061 (8)	−0.0150 (9)	0.0015 (9)
O17	0.0734 (10)	0.0636 (10)	0.0683 (10)	−0.0249 (8)	0.0213 (8)	−0.0288 (8)
O24	0.0923 (11)	0.0372 (8)	0.0528 (9)	0.0021 (7)	−0.0004 (8)	−0.0066 (7)

### Geometric parameters (Å, º)

**Table:**

C1—C5	1.499 (3)	C16—H16A	0.9300
C1—C4	1.569 (3)	C17—O17	1.217 (2)
C1—C2	1.580 (3)	C17—C18	1.491 (3)
C1—H1A	0.9800	C18—C19	1.383 (3)
C2—C11	1.514 (2)	C18—C23	1.384 (3)
C2—C3	1.542 (2)	C19—C20	1.375 (3)
C2—H2A	0.9800	C19—H19A	0.9300
C3—C17	1.519 (3)	C20—C21	1.371 (4)
C3—C4	1.557 (3)	C20—H20A	0.9300
C3—H3A	0.9800	C21—C22	1.368 (4)
C4—C24	1.504 (3)	C21—H21A	0.9300
C4—H4A	0.9800	C22—C23	1.387 (3)
C5—C10	1.390 (3)	C22—H22A	0.9300
C5—C6	1.397 (3)	C23—H23A	0.9300
C6—C7	1.380 (3)	C24—O24	1.217 (2)
C6—N6	1.469 (4)	C24—C25	1.483 (3)
C7—C8	1.359 (5)	C25—C26	1.385 (3)
C7—H7A	0.9300	C25—C30	1.387 (3)
C8—C9	1.372 (4)	C26—C27	1.370 (3)
C8—H8A	0.9300	C26—H26A	0.9300
C9—C10	1.376 (3)	C27—C28	1.369 (4)
C9—H9A	0.9300	C27—H27A	0.9300
C10—H10A	0.9300	C28—C29	1.363 (4)
C11—C16	1.383 (3)	C28—H28A	0.9300
C11—C12	1.401 (3)	C29—C30	1.383 (3)
C12—C13	1.393 (3)	C29—H29A	0.9300
C12—N12	1.460 (3)	C30—H30A	0.9300
C13—C14	1.360 (3)	N6—O1B	1.09 (3)
C13—H13A	0.9300	N6—O1A	1.192 (4)
C14—C15	1.369 (3)	N6—O2A	1.238 (4)
C14—H14A	0.9300	N6—O2B	1.35 (2)
C15—C16	1.384 (3)	N12—O3	1.198 (2)
C15—H15A	0.9300	N12—O4	1.198 (2)
			
C5—C1—C4	117.35 (16)	C16—C15—H15A	119.7
C5—C1—C2	117.82 (14)	C11—C16—C15	123.0 (2)
C4—C1—C2	88.90 (13)	C11—C16—H16A	118.5
C5—C1—H1A	110.4	C15—C16—H16A	118.5
C4—C1—H1A	110.4	O17—C17—C18	120.32 (18)
C2—C1—H1A	110.4	O17—C17—C3	119.49 (17)
C11—C2—C3	119.52 (15)	C18—C17—C3	119.87 (17)
C11—C2—C1	118.78 (14)	C19—C18—C23	118.94 (19)
C3—C2—C1	90.19 (13)	C19—C18—C17	118.30 (19)
C11—C2—H2A	109.0	C23—C18—C17	122.71 (18)
C3—C2—H2A	109.0	C20—C19—C18	120.4 (2)
C1—C2—H2A	109.0	C20—C19—H19A	119.8
C17—C3—C2	114.65 (15)	C18—C19—H19A	119.8
C17—C3—C4	122.24 (15)	C21—C20—C19	120.4 (2)
C2—C3—C4	90.76 (13)	C21—C20—H20A	119.8
C17—C3—H3A	109.2	C19—C20—H20A	119.8
C2—C3—H3A	109.2	C22—C21—C20	119.9 (2)
C4—C3—H3A	109.2	C22—C21—H21A	120.0
C24—C4—C3	119.06 (15)	C20—C21—H21A	120.0
C24—C4—C1	110.24 (15)	C21—C22—C23	120.2 (2)
C3—C4—C1	90.03 (13)	C21—C22—H22A	119.9
C24—C4—H4A	111.9	C23—C22—H22A	119.9
C3—C4—H4A	111.9	C18—C23—C22	120.1 (2)
C1—C4—H4A	111.9	C18—C23—H23A	119.9
C10—C5—C6	115.99 (19)	C22—C23—H23A	119.9
C10—C5—C1	119.59 (18)	O24—C24—C25	121.40 (17)
C6—C5—C1	124.18 (19)	O24—C24—C4	119.91 (17)
C7—C6—C5	122.2 (3)	C25—C24—C4	118.55 (16)
C7—C6—N6	116.4 (2)	C26—C25—C30	118.15 (19)
C5—C6—N6	121.5 (2)	C26—C25—C24	119.69 (19)
C8—C7—C6	119.8 (3)	C30—C25—C24	122.16 (18)
C8—C7—H7A	120.1	C27—C26—C25	121.0 (2)
C6—C7—H7A	120.1	C27—C26—H26A	119.5
C7—C8—C9	120.1 (2)	C25—C26—H26A	119.5
C7—C8—H8A	120.0	C28—C27—C26	120.0 (2)
C9—C8—H8A	120.0	C28—C27—H27A	120.0
C8—C9—C10	120.0 (3)	C26—C27—H27A	120.0
C8—C9—H9A	120.0	C29—C28—C27	120.2 (2)
C10—C9—H9A	120.0	C29—C28—H28A	119.9
C9—C10—C5	122.0 (2)	C27—C28—H28A	119.9
C9—C10—H10A	119.0	C28—C29—C30	120.1 (2)
C5—C10—H10A	119.0	C28—C29—H29A	120.0
C16—C11—C12	114.61 (17)	C30—C29—H29A	120.0
C16—C11—C2	120.86 (16)	C29—C30—C25	120.5 (2)
C12—C11—C2	124.52 (17)	C29—C30—H30A	119.8
C13—C12—C11	122.70 (19)	C25—C30—H30A	119.8
C13—C12—N12	115.67 (17)	O1A—N6—O2A	124.8 (3)
C11—C12—N12	121.63 (17)	O1B—N6—O2B	114 (2)
C14—C13—C12	120.2 (2)	O1B—N6—C6	129.1 (15)
C14—C13—H13A	119.9	O1A—N6—C6	119.5 (3)
C12—C13—H13A	119.9	O2A—N6—C6	115.7 (3)
C13—C14—C15	119.0 (2)	O2B—N6—C6	111.4 (13)
C13—C14—H14A	120.5	O3—N12—O4	120.1 (2)
C15—C14—H14A	120.5	O3—N12—C12	119.1 (2)
C14—C15—C16	120.5 (2)	O4—N12—C12	120.80 (17)
C14—C15—H15A	119.7		
			
C5—C1—C2—C11	−6.2 (2)	C14—C15—C16—C11	−1.3 (4)
C4—C1—C2—C11	−126.84 (16)	C2—C3—C17—O17	8.2 (3)
C5—C1—C2—C3	118.12 (17)	C4—C3—C17—O17	116.0 (2)
C4—C1—C2—C3	−2.51 (13)	C2—C3—C17—C18	−178.33 (15)
C11—C2—C3—C17	−107.37 (19)	C4—C3—C17—C18	−70.5 (2)
C1—C2—C3—C17	128.91 (15)	O17—C17—C18—C19	−0.5 (3)
C11—C2—C3—C4	126.25 (17)	C3—C17—C18—C19	−173.92 (18)
C1—C2—C3—C4	2.53 (13)	O17—C17—C18—C23	176.93 (19)
C17—C3—C4—C24	−9.3 (3)	C3—C17—C18—C23	3.5 (3)
C2—C3—C4—C24	110.78 (17)	C23—C18—C19—C20	−0.4 (3)
C17—C3—C4—C1	−122.65 (18)	C17—C18—C19—C20	177.1 (2)
C2—C3—C4—C1	−2.55 (13)	C18—C19—C20—C21	0.3 (4)
C5—C1—C4—C24	120.25 (17)	C19—C20—C21—C22	−0.1 (4)
C2—C1—C4—C24	−118.70 (15)	C20—C21—C22—C23	0.0 (4)
C5—C1—C4—C3	−118.56 (16)	C19—C18—C23—C22	0.4 (3)
C2—C1—C4—C3	2.49 (13)	C17—C18—C23—C22	−177.02 (18)
C4—C1—C5—C10	50.3 (2)	C21—C22—C23—C18	−0.2 (3)
C2—C1—C5—C10	−54.1 (2)	C3—C4—C24—O24	−10.5 (3)
C4—C1—C5—C6	−135.5 (2)	C1—C4—C24—O24	91.3 (2)
C2—C1—C5—C6	120.1 (2)	C3—C4—C24—C25	173.63 (16)
C10—C5—C6—C7	1.4 (4)	C1—C4—C24—C25	−84.5 (2)
C1—C5—C6—C7	−173.0 (2)	O24—C24—C25—C26	−3.1 (3)
C10—C5—C6—N6	−178.7 (2)	C4—C24—C25—C26	172.72 (19)
C1—C5—C6—N6	7.0 (4)	O24—C24—C25—C30	177.2 (2)
C5—C6—C7—C8	−0.5 (5)	C4—C24—C25—C30	−7.0 (3)
N6—C6—C7—C8	179.5 (3)	C30—C25—C26—C27	−0.4 (3)
C6—C7—C8—C9	−0.7 (5)	C24—C25—C26—C27	179.9 (2)
C7—C8—C9—C10	1.0 (5)	C25—C26—C27—C28	0.3 (4)
C8—C9—C10—C5	−0.1 (4)	C26—C27—C28—C29	0.0 (4)
C6—C5—C10—C9	−1.1 (3)	C27—C28—C29—C30	−0.3 (5)
C1—C5—C10—C9	173.6 (2)	C28—C29—C30—C25	0.1 (4)
C3—C2—C11—C16	5.1 (3)	C26—C25—C30—C29	0.2 (3)
C1—C2—C11—C16	113.4 (2)	C24—C25—C30—C29	179.9 (2)
C3—C2—C11—C12	−173.80 (17)	C7—C6—N6—O1B	153 (4)
C1—C2—C11—C12	−65.4 (2)	C5—C6—N6—O1B	−27 (4)
C16—C11—C12—C13	−1.4 (3)	C7—C6—N6—O1A	−137.9 (4)
C2—C11—C12—C13	177.54 (18)	C5—C6—N6—O1A	42.2 (5)
C16—C11—C12—N12	178.28 (18)	C7—C6—N6—O2A	41.9 (4)
C2—C11—C12—N12	−2.8 (3)	C5—C6—N6—O2A	−138.0 (3)
C11—C12—C13—C14	0.5 (3)	C7—C6—N6—O2B	−56 (2)
N12—C12—C13—C14	−179.2 (2)	C5—C6—N6—O2B	124 (2)
C12—C13—C14—C15	0.1 (3)	C13—C12—N12—O3	−9.5 (3)
C13—C14—C15—C16	0.2 (4)	C11—C12—N12—O3	170.8 (3)
C12—C11—C16—C15	1.8 (3)	C13—C12—N12—O4	169.5 (2)
C2—C11—C16—C15	−177.2 (2)	C11—C12—N12—O4	−10.2 (3)
